# High-Sensitivity Cardiac Troponin T: Risk Stratification Tool in Patients with Symptoms of Chest Discomfort

**DOI:** 10.1371/journal.pone.0035059

**Published:** 2012-04-25

**Authors:** Alma M. Mingels, Ivo A. Joosen, Mathijs O. Versteylen, Eduard M. Laufer, Mark H. Winkens, Joachim E. Wildberger, Marja P. Van Dieijen-Visser, Leonard Hofstra

**Affiliations:** 1 Department of Clinical Chemistry, Maastricht University Medical Center, Maastricht, The Netherlands; 2 Department of Cardiology, Cardiovascular Research Institute Maastricht, Maastricht University Medical Center, Maastricht, The Netherlands; 3 Department of Radiology, Cardiovascular Research Institute Maastricht, Maastricht University Medical Center, Maastricht, The Netherlands; 4 Cardiology Center Netherlands, Utrecht, The Netherlands; Sapienza University of Rome, Italy

## Abstract

**Background:**

Recent studies have demonstrated the association between increased concentrations of high-sensitivity cardiac troponin T (hs-cTnT) and the incidence of myocardial infarction, heart failure, and mortality. However, most prognostic studies to date focus on the value of hs-cTnT in the elderly or general population. The value of hs-cTnT in symptomatic patients visiting the outpatient department remains unclear. The aim of this study was to investigate the prognostic value of hs-cTnT as a biomarker in patients with symptoms of chest discomfort suspected for coronary artery disease and to assess its additional value in combination with other risk stratification tools in predicting cardiac events.

**Methods:**

We studied 1,088 patients (follow-up 2.2±0.8 years) with chest discomfort who underwent coronary calcium scoring and coronary CT-angiography. Traditional cardiovascular risk factors and concentrations of hs-cTnT, N-terminal pro-brain-type natriuretic peptide (NT-proBNP) and high-sensitivity C-reactive protein (hsCRP) were assessed. Study endpoint was the occurrence of late coronary revascularization (>90 days), acute coronary syndrome, and cardiac mortality.

**Results:**

Hs-cTnT was a significant predictor for the composite endpoint (highest quartile [Q4]>6.7 ng/L, HR 3.55; 95%CI 1.88–6.70; *P*<0.001). Survival analysis showed that hs-cTnT had significant predictive value on top of current risk stratification tools (Chi-square change *P*<0.01). In patients with hs-cTnT in Q4 versus <Q4, a 2- to 3-fold increase in cardiovascular risk was noticed, either when corrected for high or low Framingham risk score, coronary calcium scoring, or CT-angiography assessment (HR 3.11; 2.73; 2.47; respectively; all *P*<0.01). This was not the case for hsCRP and NT-proBNP.

**Conclusions:**

Hs-cTnT is a useful prognostic biomarker in patients with chest discomfort suspected for coronary artery disease. In addition, hs-cTnT was an independent predictor for cardiac events when corrected for cardiovascular risk profiling, calcium score and CT-angiography results.

## Introduction

Identification of patients at risk for acute cardiovascular events remains a challenge. One promising avenue to improve the identification of these patients is the use of serum biomarkers, which could provide a relatively easy and cost-effective step in risk stratification. Several biomarkers have been evaluated with respect to their incremental diagnostic and prognostic value [1], [2]. Elevated concentrations of high-sensitivity C-reactive protein (hsCRP), an inflammatory biomarker, are associated with future cardiovascular events, which supports the hypothesis that atherothrombosis is partly an inflammatory disease [3]. Elevated concentrations of N-terminal pro-B-type natriuretic peptide (NT-proBNP), the inactive fragment from BNP which is secreted by the cardiomyocytes in response to ventricular wall stretch, have also been associated with an increased risk of death and cardiovascular events [4]. However, none of these biomarkers have achieved widespread acceptance in daily practise as a risk stratification tool for the detection of coronary artery disease (CAD).

With the development of more accurate high-sensitivity cardiac troponin (hs-cTn) assays, new possibilities become available to improve risk stratification [5]–[7]. Recently, we demonstrated the association between hs-cTnT and CAD, as determined by coronary computed tomographic angiography (CCTA), in patients with stable chest pain [8]. We found that even mild CAD is associated with quantifiable circulating levels of hs-cTnT, which was confirmed by others [9]. This could be the result of episodes of cardiac ischemia due to a mismatch between metabolic demand and supply. An alternative mechanism could be that cardiac troponin T release is the result of dislodgement of small localized thrombi, causing micro-injury in small coronary vessels. Recently, a number of studies were published, which focused on the prognostic value of hs-cTnT. Most of these studies were performed in the elderly or the general population [10]–[13]. Less is known about the possible incremental value of hs-cTnT on top of existing risk stratification tools in patients visiting the cardiology outpatient department because of symptoms of chest discomfort suspected for CAD.

In the present study, we investigated the prognostic value of hs-cTnT in symptomatic patients with suspected CAD, and assessed its additional value in combination with other risk stratification tools in predicting cardiac events. As a comparison, we also studied hsCRP and NT-proBNP.

## Methods

### Ethics statement

This study complies with the Declaration of Helsinki and all patients gave written informed consent. The study was approved by the Institutional Review Board and Ethics Committee at the Maastricht University.

### Study population

We studied 1,114 patients with symptoms of chest discomfort who were referred from the cardiology outpatient department for CCTA because of suspected CAD, according to the appropriateness criteria for cardiac computed tomography [14]. All scans were performed in our university medical center between 2007 and 2009. Part of this population was studied previously [8].

**Figure 1:**
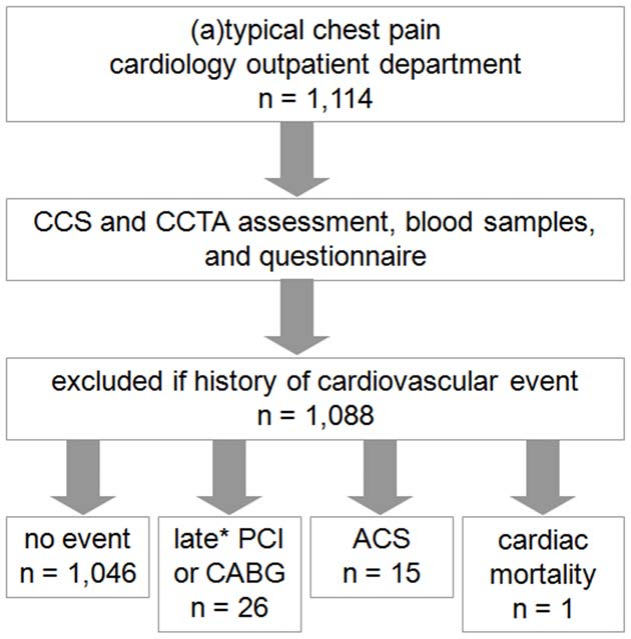
Flowchart of the study design. * Early revascularizations within 90 days after CCTA were censored at the time of PCI or CABG.

Included were patients with a recent history of chest discomfort symptoms in the presence of additional cardiovascular risk factors and/or inconclusive diagnostic test results, resulting in a population with an intermediate pretest probability of CAD. Excluded were eight patients with missing data regarding their cardiovascular risk profile and eighteen patients with a history of proven CAD, acute myocardial infarction (AMI), percutaneous coronary intervention (PCI) or coronary artery bypass grafting (CABG). The remaining 1,088 patients were subject of this study (Figure 1). Patients who were referred from the emergency department for CCTA because of acute chest pain, suspect for an acute coronary syndrome (ACS), were not included in this study. In general, patients with an allergy to iodinated contrast agent, pregnant patients and patients with an impaired renal function (defined as an estimated glomerular filtration rate <45 mL/min/1.73 m^2^) were ineligible for CCTA.

### Cardiovascular risk factors

Cardiovascular risk factors were prospectively gathered in order to calculate the Framingham risk score (FRS). The FRS is used to estimate the 10-year risk of suffering a myocardial infarction or cardiovascular death, based on age, gender, diabetes mellitus, smoking, systolic blood pressure, total cholesterol, and high-density lipoprotein (HDL) [15]. Patients were classified as smokers if they had smoked in the 12 weeks before CCTA. A positive family history was defined as having a first-degree relative with a history of myocardial infarction or sudden cardiac death before the age of sixty.

### Echocardiography acquisition

Echocardiography (Sonos 5500, Hewlett Packard, Palo Alto, CA, USA) was performed in 612 patients, using Xcelera software (Philips Healthcare, Best, the Netherlands). The left ventricular ejection fraction (LVEF) was assessed using 2D echo images. To assess the presence of left ventricular hypertrophy (LVH), we used three parameters: interventricular septum end-diastolic wall thickness (IVSEDWT), posterior wall end-diastolic wall thickness (PWEDWT) and left ventricular mass (LVMASS). Measurement of the IVSEDWT and PWEDWT were performed in the parasternal long axis.

### Coronary CT-angiography acquisition

CT-scans were performed in all patients using a 64-slice multidetector-row CT-scanner (Brilliance 64, Philips Healthcare) with a 64×0.625-mm slice collimation, a gantry rotation time of 420 ms and a tube voltage of 80–120 kV, depending on the patient's height and weight. Patients received 50 mg metoprolol tartrate orally, two hours before CCTA, to lower the heart rate (HR). When the HR was >65 beats per minute (bpm), 5–20 mg metoprolol tartrate (AstraZeneca, Zoetermeer, the Netherlands) was administered intravenously. All patients received 0.8 mg nitroglycerin spray (Pohl-Boskamp, Hohenlockstedt, Germany). HR and ECG were continuously monitored.

A non-enhanced scan was performed to determine the CCS using the Agatston method [16]. Subsequently, CCTA was performed using 85–110 mL contrast agent (Xenetix 350, Guerbet, Roissy CdG Cedex, France), which was injected in the antecubital vein with a flow rate of 6.0 mL/s, directly followed by 40 mL saline (6.0 mL/s) using a dual-head power injector. In patients with a stable HR<65 bpm, a prospective ECG-gated ‘step and shoot’ protocol was used (radiation dose 3.5±1.2 mSv). In patients with a HR >65 bpm, a retrospective ECG-gated ‘helical’ protocol with dose modulation was used (radiation dose 11.8±3.6 mSv).

### Coronary plaque assessment

All scans were independently analyzed by two cardiologists, both with level III expertise in coronary CT-angiography and blinded for patient details, using source images in Cardiac Comprehensive Analysis software (Philips Healthcare). In case of disagreement, consensus was reached by reviewing findings jointly.

CCS was expressed as Agatston score using calcium scoring software (Philips Healthcare) with a threshold of 130 Hounsfield units. The coronary tree was analyzed for the presence and severity of CAD, according to the 16-segment classification of the American Heart Association [17]. The extent of CAD was classified as absent, mild (<50% luminal stenosis), moderate (50–70% luminal stenosis) or severe (≥70% luminal stenosis), according to the guidelines of the Society of Cardiovascular Computed Tomography [18].

### Biomarker measurement

Samples were collected just before the scan, processed within two hours, and stored at −80°C until analysis. Total cholesterol, HDL and triglycerides concentrations were measured using the Synchron LX20 (Beckman Coulter Inc., Brea, CA, USA). Low-density lipoprotein (LDL) was calculated using the Friedewald equation [19] except for subjects with triglycerides >400 mg/dL and total cholesterol <50 mg/dL, in which case LDL was determined on the Cobas Mira Plus (Roche Diagnostics, Basel, Switzerland). HsCRP was measured on the BN ProSpec using the CardioPhase hsCRP assay (Siemens Diagnostics, Deerfield, IL, USA). Hs-cTnT (high sensitivity fifth generation cTnT assay) and NT-proBNP were measured on the Elecsys 2010 (Roche Diagnostics).

### Study endpoint and follow-up

The composite study endpoint was the occurrence of revascularization (PCI/CABG) >90 days after CCTA, cardiac mortality and ACS, including myocardial infarction and unstable angina requiring hospitalization. ACS was defined as typical angina pectoris, troponin T elevation (>0.01 µg/L) and ST-segment elevation/depression of ≥1 mm, or at least two of these characteristics together with invasive angiographic confirmation of a culprit lesion [20]. So, this means that patients with only troponin T elevation did not meet the criteria for an ACS. We censored follow-up when revascularization was performed within 90 days and after occurrence of the study endpoint. Patients were seen by their cardiologist on a regular basis, and all hospital visits, both outpatient department visits as well as emergency room visits, were recorded in the electronic patient records. Additionally, the national mortality records were checked. None of the attending clinicians had access to the results of the hs-cTnT, hsCRP and NT-proBNP measurements.

### Statistical analysis

To test for differences in baseline patient characteristics, we used the Pearson χ^2^ test for discrete variables and the *t*-test for continuous variables. Logistic regression and survival analysis were used to study prediction of the composite endpoint of late revascularization procedures (PCI/CABG), ACS, and cardiac mortality. Confounding was considered for baseline characteristics that differed significantly between the event group and non-event group. For Kaplan-Meier analysis, categories of independent variables were compared using the log-rank test. Cox proportional hazard regression was validated for proportionality using log-minus-log and for time dependency. It was used to evaluate the additive value of the cardiac biomarkers, based on the Chi-square change (−2 log likelihood ratio) and whether biomarkers remained significant predictors. Biomarker concentrations less than the limit of detection were set equal to the limit of detection. The threshold for statistical significance was *P*<0.05, two-sided unless stated otherwise. All data were analyzed using SPSS Statistics 18.0 (SPSS Inc., Chicago, IL, USA).

## Results

The baseline characteristics of the 1,088 patients who met the inclusion and exclusion criteria are presented in Table 1. Follow-up information was available for all patients (mean follow-up time 2.2±0.8 years). As a result of CCTA, 50 patients underwent (early) revascularization within 90 days (35× PCI, 15× CABG) and these were censored at the time of revascularization. Final survival analysis included a total of 42 patients who suffered a cardiac event: 26 patients underwent (late) revascularization >90 days after CCTA (20× PCI and 6× CABG), 15 patients suffered an ACS (6× AMI and 9× unstable angina requiring hospitalization), and one patient died due to heart failure. The overall cardiac event rate was 4%.

**Table 1:** Baseline characteristics of the study population.

Baseline characteristics	All Participants	No Event	Cardiac Event *	P value
	(n = 1,088)	(n = 1,046)	(n = 42)	
Age, mean (SD), years	56 (11)	56 (11)	59 (11)	0.067
Male gender, %	53.8	53.6	57.1	0.655
Systolic BP, mean (SD), mmHg	142 (19)	141 (19)	149 (16)	0.010
Smoking, %	26.5	25.6	50.0	0.001
Diabetes mellitus, %	8.4	8.3	12.5	0.347
Positive family history, %	39.7	39.2	51.2	0.123
Framingham risk score, median (IQR)	16.7 (9.3–27.2)	16.4 (9.2–26.4)	25.1 (14.6–48.1)	<0.001
Total cholesterol, mean (SD), mg/dL	206.1 (46.3)	205.7 (46.2)	215.7 (47.1)	0.179
LDL-C, mean (SD), mg/dL	127.6 (41.4)	127.3 (41.5)	134.0 (40.5)	0.303
HDL-C, mean (SD), mg/dL	50.4 (29.9)	50.5 (30.3)	47.9 (16.0)	0.581
Triglycerides, mean (SD), mg/dL	153.2 (102.6)	152.6 (102.9)	166.5 (93.3)	0.391
hsCRP, median (IQR), mg/dL	0.14 (0.07–0.31)	0.14 (0.07–0.31)	0.16 (0.10–0.48)	0.079
hs-cTnT, 5th generation assay, median (IQR), pg/mL	4.1 (<3.0–6.7)	4.0 (<3.0–6.6)	6.8 (<3.0–10.3)	0.015
NT-proBNP, median (IQR), pg/mL	75.5 (34.3–153.2)	74.2 (33.4–155.4)	92.3 (55.2–136.6)	0.218
LVEF, mean (SD), % †	60.3 (7.8)	60.4 (7.7)	57.2 (9.4)	0.048
IVSEDWT, mean (SD), mm †	8.9 (1.7)	8.9 (1.7)	9.5 (1.7)	0.103
PWEDWT, mean (SD), mm †	8.7 (1.1)	8.7 (1.1)	9.3 (1.4)	0.060
LVMASS, mean (SD), gram †	185 (55)	185 (54)	198 (72)	0.252
Calcium score, median (IQR)	7 (0–122)	6 (0–110)	252 (8–644)	<0.001
CCTA luminal stenosis, %				<0.001
No CAD	36.8	37.9	9.5	
Mild CAD (<50%)	38.1	39.0	16.7	
Moderate CAD (50–70%)	14.5	14.0	28.6	
Severe CAD (>70%)	10.5	9.2	45.2	

* Cardiac events: PCI>90 days, CABG>90 days, ACS, cardiac mortality.

† n = 612 underwent echocardiography.

BP, blood pressure; LDL-C, low-density lipoprotein cholesterol; HDL-C, high-density lipoprotein cholesterol; hsCRP, high-sensitive C-reactive protein; hs-cTnT, high-sensitivity cardiac troponin T; NT-proBNP, N-terminal pro-brain-type natriuretic peptide; LVEF, left ventricular ejection fraction; IVSEDWT, interventricular septum end-diastolic wall thickness; PWEDWT, posterior wall end-diastolic wall thickness; LVMASS, left ventricular mass; CCTA, coronary CT-angiography; CAD, coronary artery disease.

Table 1 shows that patients who suffered a cardiac event consisted of significantly more smokers, had higher systolic blood pressure and FRS, higher hs-cTnT concentrations, lower LVEF, higher CCS, and more severe lesions on CCTA.

### Prognostic value of hs-cTnT

Logistic regression revealed that 1-unit increase in hs-cTnT concentration resulted in a significant increase in cardiac risk (HR 1.04, 95% CI 1.01–1.06, *P* = 0.004), in contrast to NT-proBNP (HR 1.00, 95% CI 1.00–1.00, *P* = 0.645) and hsCRP (HR 1.00, 95% CI 0.98–1.03, *P* = 0.712). Comparable results were obtained when biomarker concentrations were corrected for age and gender.

Kaplan-Meier analysis shows that hs-cTnT and NT-proBNP concentrations were both significant predictors for the occurrence of cardiac events (*P*<0.001 and *P* = 0.009, respectively), in contrast to hsCRP (*P* = 0.355). Moreover, Cox regression reveals that hs-cTnT was the only significant biomarker predicting for cardiac events, either when testing the biomarker concentrations as a continuous variable (Table 2: Model 1, 4, and 7, respectively) or when present in the highest quartile Q4 (Table 2: Model 2, 5, and 8, respectively).

**Table 2:** Cox regression analysis of cardiac biomarkers for the composite endpoint of cardiac events.

Model	Cardiac biomarker	Chi-square	P value	HR	95% CI	P value
1	hs-cTnT	28.93	<0.001	1.03	1.01–1.04	<0.001
2	hs-cTnT in Q4 (>6.7 ng/L) *	17.30	<0.001	3.55	1.88–6.70	<0.001
3	hs-cTnT>URL (14 ng/L) * ^,^ †	1.08	0.299	1.85	0.57–6.02	0.307
4	NT-proBNP	0.84	0.359	1.00	1.00–1.00	0.380
5	NT-proBNP in Q4 (>18 pmol/L) *	0.72	0.396	0.70	0.31–1.60	0.399
6	NT-proBNP>URL (36 pmol/L) * ^,^ †	0.47	0.492	0.66	0.20–2.16	0.495
7	hsCRP	0.11	0.742	1.00	0.98–1.02	0.746
8	hsCRP in Q4 (>3.1 mg/L) *	0.86	0.355	1.38	0.70–2.73	0.357
9	hsCRP>URL (3 mg/L) * ^,^ †	0.61	0.436	1.31	0.66–2.60	0.437

* Dichotomous variable (yes or no); Q4, fourth quartile.

† URL = upper reference limit (used for diagnosis).

hs-cTnT, high-sensitivity cardiac troponin T; NT-proBNP, N-terminal pro-brain-type natriuretic peptide; hsCRP, high-sensitive C-reactive protein.

### Additional value of hs-cTnT on top of FRS

**Figure 2:**
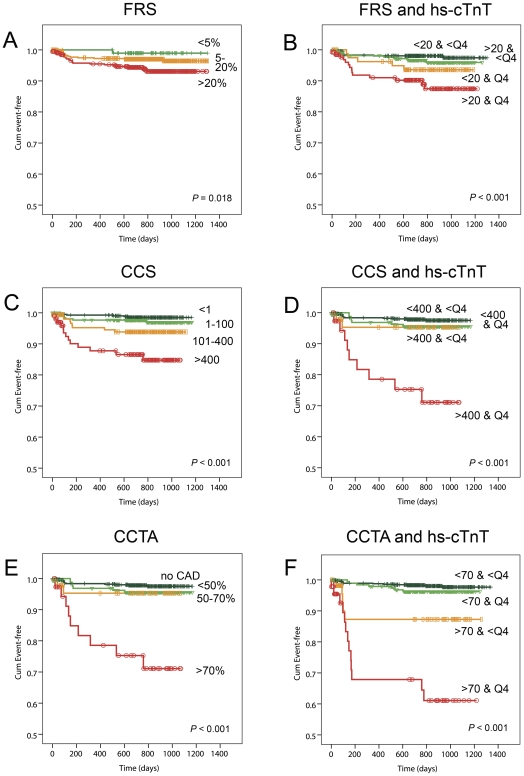
Kaplan-Meier analyses illustrating improved classification by including hs-cTnT to current risk stratification tools. Figures on the left shows Kaplan-Meier curves for FRS (A), CCS (C) and CCTA assessment (E). Figures on the right shows Kaplan-Meier curves when hs-cTnT was added to FRS (B), CCS (D) and CCTA assessment (F). Q4 = fourth quartile of hs-cTnT concentrations.

Clinical risk profiling using FRS predicted significantly for the occurrence of cardiac events (Table 1, *P*<0.001). This was confirmed using logistic and Cox regression for FRS as a continuous variable or when categorized as follows: <5% (low risk), 5–20% (intermediate risk), >20% (high risk) (all *P*<0.05). Kaplan-Meier analysis confirmed this relation, as shown in Figure 2A (*P* = 0.018). Noticeably, almost no cardiac events were observed in patients with FRS<5%.

When regarding time to event using Cox regression, hs-cTnT concentrations were predictive on top of FRS (Table 3, without versus with hs-cTnT). This was true when hs-cTnT was added to the individual parameters of the FRS (Model 1: HR hs-cTnT 1.02, *P* = 0.007 and Chi-square change 5.23, *P* = 0.022) or marginally significant when added to the complete FRS algorithm (Model 2: HR hs-cTnT 1.02, *P* = 0.018 and Chi-square change 3.58, P = 0.058). To further illustrate, we noticed a 3-fold increase in cardiac risk in patients with hs-cTnT concentrations in Q4 as compared to <Q4, independent from high or low FRS (cut-off 20%) (Model 3: HR hs-cTnT 3.11, *P* = 0.001 and Chi-square change 10.56, *P* = 0.001). In patients with FRS<20%, the cardiac event rate increased from 2.1% to 5.9% when hs-cTnT concentrations were in Q4 compared to <Q4. In patients with FRS>20%, the cardiac event rate increased from 3.6% to 10.6%, respectively. This is also illustrated using Kaplan-Meier analysis in Figure 2B (*P* = 0.001). In contrast, no significant additional value was found for NT-proBNP and hsCRP.

**Table 3:** Cox regression analysis of Framingham risk profiling for the composite endpoint of cardiac events.

Models	Without hs-cTnT			With hs-cTnT		
	HR	95% CI	P value	HR	95% CI	P value
***Model 1***						
Age	1.03	1.00–1.07	0.074	1.02	0.98–1.06	0.332
Male gender *	1.26	0.61–2.63	0.531	1.12	0.53–2.36	0.770
Total cholesterol	1.11	0.84–1.47	0.468	1.16	0.87–1.54	0.308
HDL cholesterol	0.92	0.45–1.88	0.812	0.92	0.44–1.88	0.811
Systolic blood pressure	1.02	1.00–1.03	0.071	1.02	1.00–1.04	0.030
Smoking *	3.73	1.83–7.60	<0.001	3.34	1.62–6.92	0.001
Diabetes mellitus *	1.32	0.44–3.94	0.618	0.95	0.27–3.35	0.939
hs-cTnT	-	-	-	1.02	1.01–1.04	0.007
***Model 2***						
Framingham	1.03	1.02–1.04	<0.001	1.03	1.01–1.04	<0.001
hs-cTnT	-	-	-	1.02	1.00–1.03	0.018
***Model 3***						
Framingham >20% *	2.33	1.19–4.55	0.013	1.79	0.89–3.57	0.101
hs-cTnT in Q4 *	-	-	-	3.11	1.58–6.11	0.001

* Dichotomous variable (yes or no). HDL, high-density lipoprotein; hs-cTnT, high-sensitivity cardiac troponin T; Q4, fourth quartile.

Finally, Table 1 shows that LVEF was significantly lower in the cardiac event group as compared to the non-event group (*P* = 0.048). Cox regression confirmed LVEF as a significant predictor (HR 0.96, 95% CI 0.93–1.00, *P* = 0.049). Nevertheless, the predictive value of hs-cTnT remained significant when corrected for LVEF (HR hs-cTnT 1.02, 95% CI 1.01–1.04, *P* = 0.002 and HR LVEF 0.97, 95% CI 0.93–1.01, *P* = 0.124). When LVEF was added to risk profiling as presented in Table S1 (supplemental data), smoking (Model without hs-cTnT) or smoking and hs-cTnT (Model with hs-cTnT) remained the only significant predictors.

### Additional value of hs-cTnT on top of CCS and CCTA

Kaplan-Meier analysis shows an apparent gradient of adverse survival for more severe CAD (Figure 2C and 2E). Furthermore, hs-cTnT concentrations (median, IQR) were significantly higher in mild (4.2 pg/mL, <3.0–7.3), moderate (4.7 pg/mL, <3.0–7.3), and severe CAD (6.5 pg/mL, 3.6–9.4) as compared to patients without CAD (3.3 pg/mL, <3.0–5.3), all *P*<0.001. A similar trend was found for CCS (*P*<0.001). These data show that increasing concentrations of hs-cTnT were associated with the severity of CAD, which is in line with our previous results in part of this population [8].

Cox regression in Table 4 shows the additional value of hs-cTnT on top of CAD assessment with CCS and CCTA. One unit increase in hs-cTnT resulted in a minor increase in cardiac risk (Model 1 CCS: HR hs-cTnT 1.02, *P* = 0.006 and Chi-square change 4.23, *P* = 0.040; Model 3 CCTA: HR hs-cTnT 1.01, *P* = 0.028 and Chi-square change 3.04, *P* = 0.081). To illustrate, a 2- to 3-fold increase in cardiac risk was noticed in patients with hs-cTnT concentrations in Q4, independent from high or low CCS (cut-off Agatston score 400) or luminal stenosis on CCTA (cut-off 70%) (Model 2 CCS: HR hs-cTnT 2.73, *P* = 0.007 and Chi-square change 7.20, *P* = 0.007; Model 4 CCTA: HR hs-cTnT 2.47, *P* = 0.007 and Chi-square change 7.24, *P* = 0.007. In patients with high CCS (Agatston score >400, n = 85), the cardiac event rates increased from 4.3% to 24% when hs-cTnT concentrations were in Q4 as compared to <Q4. In patients with a CCTA lesion of >70% luminal stenosis (n = 103), the cardiac event rates were 8.8% and 28% when hs-cTnT concentrations were in <Q4 and Q4, respectively. This is also illustrated using Kaplan-Meier analysis in Figure 2D and 2F for CCS and CCTA assessment, respectively (both *P* = 0.001). Again, no significant additional value was found for NT-proBNP and hsCRP.

**Table 4:** Cox regression analysis of coronary plaque assessment for the composite endpoint of cardiac events.

	Models	Without hs-cTnT			With hs-cTnT		
		HR	95% CI	P value	HR	95% CI	P value
***CCS***	***Model 1***						
	CCS	1.00	1.00–1.00	<0.001	1.00	1.00–1.00	<0.001
	hs-cTnT	-	-	-	1.02	1.01–1.04	0.006
***CCS***	***Model 2***						
	CCS>400 *	5.68	2.72–11.86	<0.001	4.53	2.13–9.64	<0.001
	hs-cTnT in Q4 *	-	-	-	2.73	1.32–5.62	0.007
***CCTA***	***Model 3***						
	Luminal stenosis on CCTA:			<0.001			<0.001
	No CAD = reference	1.00			1.00		
	<50%	1.47	0.42–5.22	0.549	1.44	0.41–5.11	0.571
	50–70%	7.25	2.27–23.11	0.001	7.09	2.22–22.62	0.001
	>70%	23.98	8.10–70.96	<0.001	21.76	7.27–65.10	<0.001
	hs-cTnT	-	-	-	1.01	1.00–1.03	0.028
***CCTA***	***Model 4***						
	luminal stenosis on CCTA>70% *	11.33	5.98–21.47	<0.001	9.23	4.79–17.82	<0.001
	hs-cTnT in Q4 *	-	-	-	2.47	1.29–4.77	0.007

* Dichotomous variable (yes or no). CCS, coronary calcium score; CCTA, coronary CT-angiography; hs-cTnT, high-sensitivity cardiac troponin T; CAD, coronary artery disease; Q4, fourth quartile.

## Discussion

Our study shows that in patients with symptoms of chest discomfort suspected for CAD, hs-cTnT was a significant predictor for the composite endpoint of late revascularizations, ACS and cardiac mortality. Over three times as much cardiac events were found in patients with hs-cTnT concentrations in the fourth quartile (cut-off 6.7 ng/L, HR 3.55, *P*<0.001) as compared to patients with hs-cTnT concentrations in the lowest three quartiles. Moreover, survival analysis showed that hs-cTnT significantly contributed to the identification of a subgroup of patients with higher risk for cardiac events. When using traditional risk factors, smoking (HR 3.34, *P* = 0.001), hs-cTnT (HR 1.02, *P* = 0.007), and systolic blood pressure (HR 1.02, *P* = 0.030) remained the only significant predictors. Hs-cTnT remained significantly predictive independent from FRS (HR 1.02–3.11, dependent whether variables were continuous or categorized). In addition, hs-cTnT improved classification on top of the extent of CAD as assessed with CCS and CCTA. To illustrate, a 2- to 3-fold increase in cardiac risk was noticed in patients with hs-cTnT concentrations in the highest quartile, independent from high or low CCS (cut-off Agatston score 400) or luminal stenosis on CCTA (cut-off 70%) (HR 2.73 and 2.47, both *P* = 0.007).

In a previous study, Reichlin et al showed that the positive predictive value of hs-cTnT in diagnosing acute myocardial infarction was only 19% (cut-off 2 pg/mL, limit of detection) or 50% (cut-off 14 pg/mL, 99^th^ percentile of healthy reference population), while the negative predictive value was nearly perfect (99–100%, dependent on cut-off) [5]. This indicates that it is of great importance to exclude false positives before widespread introduction of hs-cTn as a risk factor. On the other hand, the present study as well as other studies has shown the adverse outcome of elevated hs-cTn on cardiovascular events [10]–[12], [21], [22]. The reference change value for hs-cTnT concentrations, that is based on biological variations in healthy individuals and analytical variations, was 58% and around 95% for the short-term (4 hours) and long-term (8 weeks), respectively [23]. There are no results reported on optimal delta cut-offs considering a longer follow-up period of years apart from the study of deFilippi et al, who recently showed that for an increase in hs-cTnT concentrations >50% over two to three years, the risk for heart failure and cardiovascular death were 1.7 and 1.8-fold, respectively [11].

Question remains what the underlying pathophysiological mechanisms of elevated hs-cTnT concentrations in these patients are. Korosoglou and colleagues concluded that the presence of non-calcified coronary plaques may result in continuous leakage of troponins, possibly due to repetitive micro-embolization of atherosclerotic debris [9]. In our study, we observed a stepwise increase in hs-cTnT concentrations with increasing atherosclerotic plaque burden which supports this explanation. Alternative explanations for troponin leakage which have been supposed are demand ischemia, myocardial ischemia (for example due to coronary vasospasm), direct myocardial damage, chronic renal insufficiency, or myocardial strain because of volume or pressure overload [24]. Other possible causes of elevated hs-cTnT concentrations could be chest trauma, strenuous exercise, pericarditis, myocarditis and cardiac amyloidosis. However, in the present study we could exclude chest trauma and strenuous exercise as causes. Furthermore, ECG findings, CCTA and echocardiography did not reveal convincing evidence for alternative diagnoses like pericarditis, myocarditis or amyloidosis. Since concentrations of NT-proBNP were not increased in our patients, we felt we could exclude digestive heart failure as cause of the increased hs-cTnT concentrations.

Recently, two papers were published which feed the thought that the identification of patients at risk for a cardiovascular event may soon become easier and more accurate using hs-cTnT [10], [11]. DeFilippi et al performed serial measures of hs-cTnT in community-dwelling older adults [11]. They found a significant association between baseline hs-cTnT concentrations, changes in hs-cTnT concentrations and the development of heart failure and cardiovascular death. De Lemos et al found an association between increased hs-cTnT and structural heart disease, especially left ventricular hypertrophy, and subsequent risk for all-cause mortality [10]. However, the study by deFilippi was focusing on elderly with a mean age above 70 years, while in the study of de Lemos the vast majority of the population (77%) consisted of patients with FRS <10%. These characteristics are not typical for the patients presenting at the cardiology outpatient department. Therefore, it is not clear from those studies to what extent hs-cTnT would be of incremental value in patients presenting with symptoms of chest discomfort at the cardiology outpatient department. Moreover, the published studies focused on left ventricular hypertrophy and heart failure, respectively. It is not inconceivable that the main cause of the elevated hs-cTnT is the presence of atherosclerosis, because it is known that the majority of patients with heart failure have underlying coronary atherosclerotic disease [25]. Moreover, hypertension is an important risk factor for atherosclerosis and also the major determinant of left ventricular hypertrophy. In this study, we show that although the predictive value of LVEF for events was significant, this did not seem to confound our results. In previous work, we demonstrated that even mild CAD is associated with increased concentrations of hs-cTnT and we suggested that hs-cTnT may become a potential serum biomarker to improve the identification of patients at risk for developing cardiovascular events [8]. There is increasing evidence that ACS may be predominantly caused by such mild stenoses [26]. On the other hand, it is known that the extent of CAD provides important prognostic information in both asymptomatic and symptomatic patients. Both high CCS and ≥50% luminal stenosis on CCTA deprive prognosis significantly [27]. In this study we show that measuring hs-cTnT provides additional value on these already strong prognostic parameters. In our opinion, these findings strengthen the hypothesis that hs-cTnT is a prognostic clinical biomarker. In addition, we provide new insights into the use of hs-cTnT, which can help the physician to better identify the patient at risk of a cardiovascular event.

This study has several limitations that merit comment. First, the follow-up period is relatively short and therefore we found relatively few events. However, the event rate which we found is comparable to other previously published large CCTA-trials. Second, clinicians were not blinded for CCTA findings. Therefore, early revascularizations (within 90 days) were censored for survival analysis. However, the knowledge of the CCTA findings could still bias the clinician's behaviour after the 90 day time period. It would be interesting to blind clinicians for CCTA results, but since CCTA is part of the diagnostic work-up in our university medical center, this would be unethical. On the other hand, none of the clinicians had access to the results of the hs-cTnT, hsCRP and NT-proBNP measurements. Third, all patients were of Western European descent. It remains uncertain whether our results can be generalized to other populations. Fourth, we performed a single hs-cTnT measurement and it remains unclear in which manner hs-cTnT varies in time. Fifth, despite the fact that invasive coronary angiography is still the golden standard for coronary artery stenosis, we are convinced that the use of CCTA gives an adequate reflection of the extent and severity of plaque burden in our study population.

In conclusion, hs-cTnT is a useful prognostic biomarker in patients with symptoms of chest discomfort suspected for CAD. Hs-cTnT is associated with the extent of CAD, assessed by CCS and CCTA, and is a significant predictor for the occurrence of a future cardiac event (late revascularization, ACS, and cardiac mortality). Even better performance was obtained when hs-cTnT concentrations were combined with Framingham risk profiling. Finally, hs-cTnT also provided additional value to the assessment of CAD by coronary computed tomography.

## Supporting Information

Table S1 **Cox regression analysis including LVEF.** Cox regression analysis of risk profiling and coronary plaque assessment including LVEF for the composite endpoint of cardiac events.
(DOC)
