# Supplementary material for: High-Sensitivity Cardiac Troponin T: Risk Stratification Tool in Patients with Symptoms of Chest Discomfort
Source: PLoS One. 2012 Apr 25;7(4):e35059. doi: 10.1371/journal.pone.0035059 (PMC3338816; doi:10.1371/journal.pone.0035059)
Supplement: Table S1 — Cox regression analysis including LVEF. Cox regression analysis of risk profiling and coronary plaque assessment including LVEF for the composite endpoint of cardiac events. (DOC) [file pone.0035059.s001.doc]

**Supplemental Data**

| **Table S1.** Cox regression analysis of risk profiling and coronary plaque assessment including LVEF for the composite endpoint of cardiac events. | | | | | | |
| --- | --- | --- | --- | --- | --- | --- |
|  |  | | |  | | |
|  | **Without hs-cTnT** | | | **With hs-cTnT** | | |
|  | **HR** | **95% CI** | **P value** | **HR** | **95% CI** | **P value** |
| **Model FRS** |  |  |  |  |  |  |
| Age | 1.03 | 0.98-1.08 | 0.249 | 1.01 | 0.96-1.06 | 0.747 |
| Male gender * | 0.82 | 0.31-2.18 | 0.697 | 0.68 | 0.25-1.90 | 0.464 |
| Total cholesterol | 1.22 | 0.86-1.74 | 0.259 | 1.32 | 0.92-1.91 | 0.135 |
| HDL cholesterol | 0.59 | 0.14-2.53 | 0.475 | 0.63 | 0.14-2.78 | 0.540 |
| Systolic blood pressure | 1.02 | 1.00-1.04 | 0.104 | 1.02 | 1.00-1.05 | 0.051 |
| Smoking * | 6.85 | 2.57-18.2 | <0.001 | 5.94 | 2.19-16.1 | <0.001 |
| Diabetes mellitus * | 2.06 | 0.58-7.38 | 0.265 | 1.35 | 0.29-6.27 | 0.700 |
| LVEF | 0.97 | 0.93-1.02 | 0.262 | 0.97 | 0.93-1.02 | 0.267 |
| hs-cTnT | - | - | - | 1.02 | 1.00-1.04 | 0.041 |
| **Model CCS** |  |  |  |  |  |  |
| CCS | 1.00 | 1.00-1.00 | 0.012 | 1.00 | 1.00-1.00 | 0.057 |
| LVEF | 0.98 | 0.93-1.02 | 0.337 | 0.99 | 0.94-1.04 | 0.653 |
| hs-cTnT | - | - | - | 1.02 | 1.00-1.04 | 0.022 |
| **Model CCTA** |  |  |  |  |  |  |
| Luminal stenosis on CCTA: |  |  | <0.001 |  |  | <0.001 |
| No CAD = reference | 1.00 |  |  | 1.00 |  |  |
| <50% | 0.45 | 0.04-4.91 | 0.509 | 0.44 | 0.04-4.84 | 0.501 |
| 50-70% | 7.07 | 1.36-36.7 | 0.020 | 7.26 | 1.40-37.7 | 0.018 |
| >70% | 29.2 | 6.58-129 | <0.001 | 27.3 | 6.10-122 | <0.001 |
| LVEF | 0.98 | 0.94-1.02 | 0.313 | 0.99 | 0.95-1.03 | 0.570 |
| hs-cTnT | - | - | - | 1.01 | 1.00-1.02 | 0.183 |
| *Dichotomous variable (yes or no). HDL, high-density lipoprotein; hs-cTnT, high-sensitivity cardiac troponin T; LVEF, left ventricular ejection fraction; Q4, fourth quartile. | | | | | | |
